# Algorithmic discrimination: examining its types and regulatory measures with emphasis on US legal practices

**DOI:** 10.3389/frai.2024.1320277

**Published:** 2024-05-21

**Authors:** Xukang Wang, Ying Cheng Wu, Xueliang Ji, Hongpeng Fu

**Affiliations:** ^1^Sage IT Consulting Group, Shanghai, China; ^2^School of Law, University of Washington, Seattle, WA, United States; ^3^Faculty of Law, The Chinese University of Hong Kong, Sha Tin, Hong Kong SAR, China; ^4^Khoury College of Computer Sciences, Northeastern University, Seattle, WA, United States

**Keywords:** algorithmic discrimination, regulatory measures, automated decision-making, computational intelligence, AI and law

## Abstract

**Introduction:**

Algorithmic decision-making systems are widely used in various sectors, including criminal justice, employment, and education. While these systems are celebrated for their potential to enhance efficiency and objectivity, they also pose risks of perpetuating and amplifying societal biases and discrimination. This paper aims to provide an indepth analysis of the types of algorithmic discrimination, exploring both the challenges and potential solutions.

**Methods:**

The methodology includes a systematic literature review, analysis of legal documents, and comparative case studies across different geographic regions and sectors. This multifaceted approach allows for a thorough exploration of the complexity of algorithmic bias and its regulation.

**Results:**

We identify five primary types of algorithmic bias: bias by algorithmic agents, discrimination based on feature selection, proxy discrimination, disparate impact, and targeted advertising. The analysis of the U.S. legal and regulatory framework reveals a landscape of principled regulations, preventive controls, consequential liability, self-regulation, and heteronomy regulation. A comparative perspective is also provided by examining the status of algorithmic fairness in the EU, Canada, Australia, and Asia.

**Conclusion:**

Real-world impacts are demonstrated through case studies focusing on criminal risk assessments and hiring algorithms, illustrating the tangible effects of algorithmic discrimination. The paper concludes with recommendations for interdisciplinary research, proactive policy development, public awareness, and ongoing monitoring to promote fairness and accountability in algorithmic decision-making. As the use of AI and automated systems expands globally, this work highlights the importance of developing comprehensive, adaptive approaches to combat algorithmic discrimination and ensure the socially responsible deployment of these powerful technologies.

## 1 Introduction

The significant influence of big data mining on human existence has emerged in recent years due to the progress of computer and information technologies. The broad application of machine learning-based algorithms to solve challenging problems has been made possible by the combination of enormous data streams with sophisticated algorithmic analysis and technological capabilities (Simmons, 2018). In many fields today, including hiring, law enforcement, education, credit reporting, criminal justice, and stock trading, algorithms are used to make decisions (Mayson, 2019). For example, algorithms are used by judges to estimate the risk of reoffending by ex-offenders (Berk et al., 2021), by education departments to decide whether to rehire teachers (Hous, n.d.), and by schools to choose whether to admit students (Swist and Gulson, 2023).

Algorithmic decision-making has led to the emergence of new and complex types of discrimination, which are frequently hidden within the algorithms, even though it can somewhat lessen the subjectivity of human judgments (Lepri et al., 2018). The ubiquity of algorithmic prejudice and the possible social, ethical, and legal problems it raises are becoming more widely recognized among academics and governmental bodies (Selbst and Barocas, 2016). Algorithmic discrimination can manifest in various forms, such as bias by the algorithmic agents, biased feature selection, preventive controls, consequential liability regulation, and big data discrimination (Kim, 2016). These different types of algorithmic bias can lead to unfair treatment and disparate impacts on protected groups, raising concerns about equal rights, due process, and social justice (Kroll et al., 2017).

In response to these challenges, governments worldwide have implemented regulatory measures to address algorithmic discrimination in society (Goodman and Flaxman, 2017). In the United States, all states and the federal government have included algorithms that have a significant discriminatory effect in their legal frameworks and started judicial evaluations of these algorithms (Simmons, 2018). Legal approaches include principled regulation, specific industry guidance, preventive controls, consequential liability, self-regulation, and heteronomy regulation (Zarsky, 2016). These regulatory strategies aim to ensure algorithmic fairness, transparency, and accountability while balancing the benefits and risks of automated decision-making systems (Doshi-Velez et al., 2017).

However, existing research primarily focuses on specific cases, lacking a comprehensive summary of the basic types of algorithmic discrimination and a systematic examination of legal regulation methods and judicial review processes (Gillis and Spiess, 2019). This paper aims to bridge this gap by studying these issues, with a particular focus on the relevant legal practices in the United States for ease of analysis. By providing a structured taxonomy of algorithmic discrimination types, evaluating current regulatory approaches, and analyzing judicial review standards, this study seeks to contribute to the ongoing discourse on algorithmic fairness and offer insights for policymakers, legal practitioners, and researchers.

The remainder of this paper is organized as follows. Section 2 discusses the methodology employed in this study. Section 3 presents a systematic review of the algorithmic discrimination literature, identifying key themes, findings, and gaps. Section 4 discusses the basic types of algorithmic discrimination, including bias by algorithmic agents, discrimination based on feature selection, proxy discrimination, disparate impact, and targeted advertising. Section 5 examines the legal regulation of algorithmic discrimination, covering principled regulation, preventive controls, consequential liability, self-regulation, and heteronomy regulation. Section 6 analyzes the judicial review processes for assessing claims of intentional and unintentional algorithmic discrimination in U.S. courts. Section 7 presents two case studies that illustrate the real-world manifestations and consequences of algorithmic discrimination. Section 8 provides a comparative analysis of algorithmic discrimination regulation in other jurisdictions, highlighting common challenges and promising practices. Finally, Section 9 concludes by summarizing the key findings, discussing their implications, and offering recommendations for future research and policy development.

## 2 Methodology

To comprehensively examine the types of algorithmic discrimination and analyze the legal regulatory measures and judicial review processes, we employed a robust, multi-method research approach. Our methodology combined a systematic literature review, legal document analysis, and comparative case studies.

First, we conducted a systematic literature review to identify the key types of algorithmic discrimination discussed in the existing scholarly work. We selected peer-reviewed articles, conference papers, and book chapters that directly addressed the types, causes, and consequences of algorithmic discrimination. The identified sources were then categorized and synthesized to develop a comprehensive taxonomy of algorithmic discrimination types.

Next, we performed a legal document analysis to examine the regulatory measures and judicial review standards related to algorithmic discrimination in the United States. We collected and analyzed relevant federal and state laws, regulations, guidelines, and court cases using legal databases such as Westlaw, LexisNexis, and Bloomberg Law. We focused on identifying the key legal principles, specific industry requirements, preventive and corrective mechanisms, and liability frameworks that govern algorithmic decision-making. The legal documents were analyzed using a qualitative content analysis approach to extract the main themes, patterns, and trends in algorithmic discrimination regulation.

Besides, we conducted comparative case studies. We selected representative cases from various domains, such as employment, criminal justice, housing, and credit, where algorithmic bias has been alleged or proven. For each case, we gathered information from court filings, judicial opinions, media reports, and other publicly available sources. We then analyzed the cases using a structured framework that examined the algorithmic systems involved, the types of discrimination alleged, the legal arguments put forth by the parties, and the outcomes or settlements reached. The case studies provide insights into the practical application of legal principles and the complexities of addressing algorithmic discrimination in different settings.

By combining a systematic literature review, legal document analysis, and comparative case studies, our methodology provided a comprehensive approach to examining the types of algorithmic discrimination and the legal responses to this emerging challenge. This multi-method strategy allowed us to develop a nuanced understanding of the current state of algorithmic bias and the effectiveness of different regulatory approaches, while also identifying areas for future research and policy development.

## 3 Systematic review of algorithmic discrimination literature

To gain a comprehensive understanding of the current state of research on algorithmic discrimination, we conducted a systematic review of the existing literature. The review aimed to identify the key themes, findings, and gaps in the scholarly work on the types, causes, consequences, and regulation of algorithmic bias.

### 3.1 Search strategy

We searched multiple academic databases, including ACM Digital Library, IEEE Xplore, LexisNexis, HeinOnline, and Google Scholar, using a combination of keywords related to algorithmic discrimination. The search query included terms such as “algorithmic discrimination,” “algorithmic bias,” “machine learning fairness,” “AI and discrimination,” and “algorithmic decision-making.” We also conducted backward and forward citation tracking of the identified articles to find additional relevant sources.

### 3.2 Results

Our search and selection process yielded a total of 85 articles that met the inclusion criteria. The articles spanned a range of disciplines, including computer science, law, social sciences, and ethics, reflecting the interdisciplinary nature of the algorithmic discrimination research.

The thematic analysis revealed several key themes in the literature:

- *Types and manifestations of algorithmic discrimination*: The articles identified various forms of algorithmic bias, such as discrimination based on biased training data (Selbst and Barocas, 2016), discriminatory feature selection (Zliobaite, 2015), proxy discrimination (Prince and Schwarcz, 2019), disparate impact (Hellman, 2020), and targeted advertising (Speicher et al., 2018). These discussions highlighted the complex and multifaceted nature of algorithmic discrimination and the need for a nuanced understanding of its different manifestations.
- *Sources and causes of algorithmic bias*: Many articles examined the underlying factors that contribute to algorithmic discrimination, such as historical biases in data (Crawford and Schultz, 2014), lack of diversity in the development teams (West et al., 2019), and the opacity of algorithmic systems (Pasquale, 2015). These analyses emphasized the importance of considering the social and historical contexts in which algorithms are designed and deployed, as well as the role of human choices and values in shaping algorithmic outcomes.
- *Legal and regulatory responses to algorithmic discrimination*: A significant portion of the literature focused on the legal and policy implications of algorithmic bias, discussing issues such as the applicability of existing anti-discrimination laws (Kim, 2016), the need for new regulations specific to algorithmic decision-making (Selbst and Powles, 2018), and the challenges of enforcing accountability and transparency in algorithmic systems (Kroll et al., 2017). The articles highlighted the limitations of current legal frameworks and the need for adaptive and proactive regulatory approaches.
- *Strategies for mitigating algorithmic bias*: Many articles proposed or evaluated various strategies for reducing algorithmic discrimination, such as pre-processing training data (Kamiran and Calders, 2012), incorporating fairness constraints into machine learning models (Zafar et al., 2017), and implementing algorithmic auditing and impact assessments (Reisman et al., 2018). These discussions underscored the importance of a multifaceted approach to bias mitigation, combining technical, organizational, and regulatory measures.
- *Interdisciplinary perspectives on algorithmic fairness*: The literature on algorithmic discrimination drew on insights from various disciplines, including computer science, law, social sciences, and ethics. Articles emphasized the need for interdisciplinary collaboration and dialogue to fully understand and address the complex challenges posed by algorithmic bias (Lepri et al., 2018).

Despite the growing literature on algorithmic discrimination, the systematic review also identified several gaps and areas for future research. These included the need for more empirical studies on the real-world impacts of algorithmic bias, the development of standardized frameworks for auditing and assessing algorithmic systems, and the exploration of participatory and community-driven approaches to algorithmic governance.

Overall, the systematic review provided a comprehensive overview of the current state of research on algorithmic discrimination, highlighting the key themes, findings, and challenges in this rapidly evolving field. The insights from the review informed our analysis of the types of algorithmic discrimination (Section 4), the legal and regulatory responses (Section 5), and the comparative perspectives (Section 8) in the subsequent sections of the paper.

## 4 Basic types of algorithmic discriminations

### 4.1 Algorithmic discrimination based on biased agents

One common type of algorithmic discrimination occurs when the decision-making process relies on biased agents or data sources. This can happen when the algorithms are trained on historical data that reflects past discriminatory practices or societal biases (Selbst and Barocas, 2016). For example, a hiring algorithm trained on past employment records may learn to discriminate against women or minorities if those groups were underrepresented or underpromoted in the training data (Ajunwa, 2019).

Another way biased agents can lead to discrimination is through the use of proxy variables that correlate with protected characteristics. For instance, an algorithm used to determine credit risk might use ZIP codes as a proxy for race, leading to discriminatory outcomes even if race is not explicitly considered (Citron and Pasquale, 2014). Biased agents can also emerge from the use of incomplete or unrepresentative data, which fails to adequately capture the diversity of the population (Bolukbasi et al., 2016).

### 4.2 Discrimination based on feature selection

Algorithmic discrimination can also arise from the way features are selected and weighted in the decision-making process. This type of discrimination is closely related to the problem of biased data, but it specifically involves the choices made by algorithm designers in determining which attributes to include and how to prioritize them (Selbst and Barocas, 2016). For example, a college admissions algorithm that heavily weights standardized test scores may discriminate against students from disadvantaged backgrounds who had less access to test preparation resources (Todolí-Signes, 2019). Similarly, a predictive policing algorithm that relies on historical crime data may perpetuate biases against communities of color that have been subject to over-policing and disproportionate arrests (Richardson et al., 2019).

Feature selection discrimination can also occur when algorithms use seemingly neutral attributes that correlate with protected characteristics. For instance, an advertising algorithm that targets users based on their web browsing history may inadvertently exclude certain demographic groups that have different online behaviors (Sweeney, 2013).

### 4.3 Discrimination through proxy variables and masked attributes

Another type of algorithmic discrimination involves the use of proxy variables or masked attributes that serve as stand-ins for protected characteristics. This can allow algorithms to engage in “redlining” or other forms of discrimination while appearing to be neutral and objective (Gillis and Spiess, 2019). For example, an algorithm used to screen job applicants might not explicitly consider race or gender, but it could use variables like “distance from workplace” or “gaps in employment history” that correlate with those protected attributes (Kim, 2016). Similarly, a facial recognition algorithm might not directly label individuals by race, but it could use skin tone or other physical features as proxies for racial classification (Buolamwini and Gebru, 2018).

The use of proxy variables can make it difficult to detect and prove discrimination, as the algorithm’s decisions may be based on seemingly neutral and objective criteria. However, the impact of these decisions can still be discriminatory if they disproportionately affect certain protected groups (Zarsky, 2014).

### 4.4 Discrimination in targeted advertising and pricing

Algorithmic discrimination can also occur in the context of targeted advertising and dynamic pricing. With the vast amounts of personal data collected by online platforms and marketers, algorithms can be used to segment consumers into granular groups and deliver customized ads or prices based on their perceived preferences, behaviors, and characteristics (Calo, 2013).

While targeted advertising can be beneficial for both consumers and businesses, it can also lead to discriminatory outcomes. For example, a job posting that is only shown to younger users or a housing ad that excludes certain racial groups would be engaging in unlawful discrimination (Speicher et al., 2018). Similarly, dynamic pricing algorithms that charge higher prices to consumers in low-income or minority neighborhoods could be violating fair lending laws (MacKay and Weinstein, 2022).

The use of algorithmic targeting and pricing can be especially problematic when it relies on sensitive personal information, such as race, gender, age, or sexual orientation. Even if this information is inferred rather than explicitly provided, its use in advertising and pricing decisions can still be discriminatory (Miller, 2015).

### 4.5 Disparate impact discrimination

Finally, algorithmic systems can engage in discrimination through disparate impact, even if they do not explicitly use protected characteristics or proxy variables. Disparate impact occurs when a facially neutral policy or practice has a disproportionate adverse effect on a protected group (Selbst and Barocas, 2016).

In the context of algorithms, disparate impact can arise from a variety of sources, including biased data, flawed feature selection, or the interaction of multiple algorithms across different domains (Selbst et al., 2019). For example, a hiring algorithm that selects candidates based on their similarity to current high-performing employees might inadvertently exclude women or minorities if the current workforce is predominantly male or white (Ajunwa, 2019).

Disparate impact discrimination can be particularly challenging to detect and address, as it does not involve explicit bias or intentional discrimination. Instead, it requires a careful analysis of the outcomes and impacts of algorithmic systems across different demographic groups (Crawford and Schultz, 2014). These basic types of algorithmic discrimination illustrate the wide range of ways in which bias and unfairness can emerge in automated decision-making systems. Understanding these different manifestations of discrimination is crucial for developing effective legal and policy responses that can promote greater algorithmic fairness and accountability.

## 5 Legal regulation of algorithmic discrimination

Monitoring and balancing this kind of “quasi-public power,” or algorithmic power, is difficult due to its lack of openness, unwillingness to answer inquiries, and lack of justifications. What is characterized as “algorithmic tyranny” consequently results from an imbalance between authority and individual rights (Lepri et al., 2017). Various regulatory measures have been put in place by governments worldwide to tackle algorithmic prejudice in society.

### 5.1 Principled regulation and specific regulation

According to the U.S. government, the traditional equal protection clause’s guiding principles can apply to both algorithmic and traditional forms of discrimination (Miller, 2015). Nonetheless, several ideas in regulation lack clarity and clear applicability. Because of the various forms of algorithmic discrimination and intricate internal mechanisms, it is difficult to create consistent institutional arrangements. Therefore, taking into account certain circumstances, targeted restrictions are required. In addition to ethical regulation, the US will create targeted regulatory actions for particular industries to combat algorithmic discrimination.

By evaluating pertinent U.S. laws and state court decisions, it can be concluded that algorithmic discrimination is governed by the equality principle, which forbids discrimination in general. It highlights the need for algorithmic designers to abide by current laws that provide equal protection against discrimination to citizens and consumers (Janssen and Kuk, 2016).

For example, U.S. laws demand that the Fair Credit Reporting Act and the Civil Rights Act be complied with when using big data and algorithms for decision-making. According to the Obama administration, authorities “should develop programs to investigate and address such discriminatory practices” and “expand their technical expertise to be able to identify big data analytics that have discriminatory effects on protected groups” (MacCarthy, 2017). Furthermore, the equal protection clause of the Fourteenth Amendment to the Constitution must be complied with by algorithms and big data analysis used for automatic decision-making.

### 5.2 Preventive controls

Preventive control is essential to curb algorithmic discrimination. It covers two main areas: algorithmic evaluation and review, and democratic data collection and exit methods. Reviewing and evaluating algorithms involves validating them with the help of professionals, policymakers, and the public to reduce bias and unintended consequences of discrimination. To ensure a regulated computer system, algorithmic review must be incorporated from the earliest stages of system design. This helps reduce the potential risk of discrimination in algorithmic decisions. For example, whether sensitive characteristics, such as race or gender, play a subtle role in decision-making should be investigated to prevent racial or gender discrimination in the decision-making process. Not only should specific categories of sensitive characteristics, such as gender and race, be defined, but they should also be scrutinized for discriminatory information. For example, the use of an individual’s zip code in conjunction with race data to determine loan eligibility can lead to unfair results, so this situation also needs to be regulated and corrected (Custers, 2013).

Algorithmic fairness also depends on the algorithm itself, not just the data. To root out algorithmic discrimination, basic data regulations must be strengthened. Data collection and exit are two aspects of basic data regulation.

To ensure that algorithmic decision-makers obtain individual consent before collecting and using personal data to make decisions, the first step in democratic data collection is to implement transparency and algorithmic disclosure mechanisms. The EU General Data Protection Regulation and the Privacy Act of the United States stipulate that data controllers must comply with existing laws to ensure that consent is obtained for personal data in the decision-making process. The UK’s Data Protection Act 2017 improves the ‘informed consent’ approach and adds new requirements for individual consent (Hamilton, 2019).

In addition, users’ interactions with tech platforms establish electronic trails that retailers and data-mining companies can use to infer user preferences and display customized ads. The European Union’s General Data Protection Regulation provides for a “right to be forgotten,” allowing data controllers to request the deletion or elimination of data after its intended use has been met. The UK’s Data Protection Act 2017 also enforces the “right to be forgotten,” enabling anyone to ask social media companies to remove any personally identifiable information they have uploaded (OECD, 2019a,b).

These preventive controls help ensure that algorithmic decisions are fair and non-discriminatory. This type of regulation not only helps reduce the potential risk of discrimination, it also provides a way for affected individuals to assert their rights. By implementing these controls in algorithms and data processing, we can better balance scientific and technological advances with legal frameworks to ensure fair and lawful decision-making.

### 5.3 Consequential liability regulation

The key to the consequence-based responsibility regulation model is to ensure that when algorithmic decisions produce discriminatory results and adversely affect the relevant people, the decision maker or user can take responsibility and take corrective measures (O’neil, 2017). The goal of this regulatory model is to prohibit discriminatory effects in algorithmic decisions by upholding equal rights, and to compensate for inequities with the repair and punishment of actual harm. More detailed information can be analyzed in the following two cases:

#### 5.3.1 Case 1: employment discrimination

In the field of employment, some companies may use algorithms to screen and evaluate candidates’ resumes and abilities. However, if these algorithms use inappropriate characteristics or data, such as sensitive information such as gender, race, or age, it can lead to discriminatory hiring decisions. In this case, the victim may choose to take legal action and file a discrimination lawsuit. In the United States, the issue of employment discrimination is usually regulated by the Civil Rights Act. Specifically, Title VII of the Civil Rights Act prohibits employers from discriminating against employees or job applicants based on race, sex, religion, age, national origin, and other factors. If an algorithmic decision is deemed to violate these laws, employers may be required to take corrective action, including reconsidering the hiring decision and compensating victims for their damages (Gillis and Spiess, 2019).

#### 5.3.2 Case 2: housing discrimination

In the housing sector, some real estate companies may use algorithms to determine rental pricing or housing allocation. If these algorithms use race, gender, or other legally protected characteristics to make decisions, they can lead to housing discrimination. Victims can choose to seek redress by filing a lawsuit.

Similarly, the Civil Rights Act in the United States applies to housing discrimination. If algorithmic decisions are deemed to have violated these laws, real estate companies may face legal liability, need to take corrective action, and possibly compensate victims for their losses (Bogen and Rieke, 2018).

These cases highlight the role of the post-event regulatory model, which seeks justice and repair through legal processes when discriminatory effects occur. This regulatory approach ensures fairness in algorithmic decisions by emphasizing equal rights, while providing a way for those who are unfairly treated to assert their rights.

### 5.4 Self-regulation and heteronomy regulation

For the purpose of standardizing operational and decision-making procedures, minimizing or eliminating the risks and negative effects of algorithm discrimination, and strengthening the formulation of basic algorithm principles, self-regulation essentially depends on industry self-control (Kim, 2017). The Association for Computing Machinery (ACM) has published seven key principles for algorithmic transparency and auditability, which are as follows:

- Awareness: All stakeholders involved in analytic systems, including owners, designers, builders, users, should be aware of the potential biases in design, implementation, and use, and the harm these biases can cause to individuals and society.
- Access and redress: Regulators should encourage the adoption of mechanisms that allow individuals and groups adversely affected by algorithmic decisions to question and seek redress.
- Accountability: Institutions should be held responsible for the decisions made by the algorithms they use, even if it is challenging to provide a detailed explanation of how the algorithms arrived at their results.
- Explanation: Systems and institutions using algorithmic decision-making are encouraged to provide explanations of both the algorithm’s procedures and specific decisions, especially in public policy contexts.
- Data Provenance: Builders of algorithms should maintain a description of how training data was collected, along with an analysis of potential biases introduced by the human or algorithmic data-gathering process. Public scrutiny of the data allows for corrections, though access may be restricted to qualified and authorized individuals due to privacy and security concerns.
- Auditability: Models, algorithms, data, and decisions should be recorded to allow auditing in cases where harm is suspected.
- Validation and Testing: Institutions should use rigorous methods to validate their models, document the methods and results, and routinely conduct tests to assess whether the model generates discriminatory harm. The results of such tests should ideally be made public (Council, US Public Policy, 2017).

The Equal Employment Opportunity Commission (EEOC) of the United States has been actively investigating labor recruitment instances involving the use of algorithms. It is forbidden to find out information regarding sexual orientation, political orientation, race, or religion during the recruitment process, even if it has no discernible effect on the hiring process (Rubinstein, 2010). From the standpoint of data privacy and personal information protection, algorithmic discrimination is governed by the U.S. Federal Trade Commission (FTC). It contends that algorithmic discrimination fundamentally infringes upon persons’ rights to personal data privacy. Under the pretext of bolstering the “informed consent” framework for privacy protection, the FTC has instituted a post-examination mechanism to prevent data privacy violations and has introduced the notion of “privacy by design” in order to combat algorithmic discrimination. Businesses must therefore incorporate “informed consent” privacy protection into their routine operations (Hacker and Petkova, 2017).

## 6 Judicial review of algorithmic discrimination

### 6.1 Review on deliberate algorithmic discrimination

In this type of censorship, whether an algorithm user is liable for discrimination depends on whether there is subjective intent or deep-rooted bias. Even if algorithms discriminate against certain aspects and cause harm, users may not be held accountable if they do not have malicious intent or deep-seated bias against discrimination. This type of review is intended to distinguish between those situations that intentionally create discrimination and those that inadvertently lead to it (Weaver, 2017).

In effect, the court distinguished two signs of intentional discrimination in algorithmic decisions:

Explicit discriminatory intent: In the first case, an algorithm user makes a decision by considering membership of a protected group and intentionally changes some aspect of the algorithm or its components to produce a biased result. In this case, algorithmic discrimination is actually just human bias masked by algorithms (Wachter, 2022). For example, if a bank intentionally denies a loan to an applicant of a certain ethnic group, even though they meet the lending criteria, it is a clear discriminatory intent.

Implicit discriminatory intent: The second category involves algorithmic users influencing their algorithmic decisions by using a bias against a protected group, but this influence may be unintentional. In this case, the user of the algorithm uses unbiased data within the framework of the algorithm, but influences the members of the protected group through their decisions (Prince and Schwarcz, 2019). For example, if an employer uses a hiring algorithm that screens resumes without regard to gender, but the algorithm still favors male applicants because historically the majority of the company’s hires have been male, this could be an implied discriminatory intent.

The goal of this type of review is to ensure that algorithm users do not intentionally introduce discrimination into their decisions, whether by tweaking the algorithm itself or by selecting specific data or parameters. By clearly defining the circumstances of intentional and unintentional discrimination, law and regulatory agencies can more effectively develop rules and penalties to reduce the adverse effects of algorithmic discrimination on individuals and society (Prinsloo et al., 2023). This helps protect the principles of fairness and equality, and encourages algorithm developers and users to be more careful with data and technology. In regulation, there is a need to weigh how to punish obvious intentional discrimination against potential implicit discrimination to ensure a more just and inclusive society (Bonsón et al., 2023).

### 6.2 No-fault algorithm discrimination review mode

In the absence of obvious intent or entrenched bias, unintentional discrimination review in algorithmic decisions is another approach that focuses on assessing the actual impact of algorithmic discrimination. Although there is no explicit intent or malice, an algorithmic decision can still be considered discrimination if it adversely affects a protected group. This approach to review aims to capture and correct unconscious discrimination that may arise in algorithmic decisions in order to uphold the principles of fairness and equality (Joamets, 2022).

Data analysis is the first step in unintentional algorithmic discrimination review. The reviewers conduct a detailed analysis of the data used in algorithmic decisions to determine if there are trends or disparities that disadvantage the protected group (Liu et al., 2023). This may involve the distribution of sensitive characteristics such as gender, race, age of the data and their impact on decision-making. By digging into the data, reviewers can determine if there are potential inequities. This is followed by an impact assessment, in which reviewers assess the actual impact of the algorithmic decision on the protected group. This includes examining the output of algorithms, such as loan approvals or career opportunity allocations, to see if there are unfair or unequal outcomes. If it is found that certain groups are being treated unfavourably, then this may be a sign of unintentional discrimination (Fan and Liu, 2022).

Cause analysis is the next step in the review. The reviewer will investigate the reasons for the possible adverse effects in the algorithmic decisions (He and Ding, 2022). This may include bias in the data source, such as inequality in historical data, the way features are selected, preferences in model selection, and other factors. By identifying potential problem sources, steps can be taken to correct discriminatory outcomes. Finally, follow-up measures may include recommendations for corrective actions to mitigate adverse effects. This includes recalibrating the algorithm, re-selecting features, re training the model, or adopting other methods to ensure that the algorithm does not produce discriminatory results. The purpose of these measures is to ensure that the algorithm does not adversely affect certain groups in practical application, even if this is not the explicit intention of the users of the algorithm (Lünich and Kieslich, 2024).

In summary, the goal of unintentional algorithmic discrimination review is to ensure that algorithmic decisions do not have an unfair or discriminatory effect, even if the algorithmic user does not have a clear intentional intent to discriminate (Holford, 2022). This helps to ensure that the principles of fairness and equality are upheld in algorithmic applications, thereby reducing adverse social impacts and enhancing trust in algorithmic decisions. In regulation, it is important to identify and correct unintentional discrimination in a timely manner in order to build more inclusive and fair computer systems (Martin and Waldman, 2023). By continually improving algorithmic review and regulatory processes, we can better address the algorithmic challenges of modern society and promote social equity and justice (Giovanola and Tiribelli, 2022).

### 6.3 Unintentional discrimination: detection, regulation, and challenges

Unintentional discrimination in algorithmic systems is a complex issue that requires careful attention and robust methods for detection and regulation. Unlike intentional discrimination, where there is a clear motive or intent to discriminate, unintentional discrimination often arises from biases embedded in the data, features, or models used by the algorithm, without any explicit discriminatory intent on the part of the developers or users (Selbst and Barocas, 2016).

Detecting unintentional discrimination poses significant challenges, as it requires a deep understanding of the algorithm’s inputs, processing, and outputs, as well as the social and historical contexts in which it operates. One key method for identifying unintentional bias is through statistical analysis of the algorithm’s outcomes, looking for patterns of disparate impact on protected groups (Kleinberg et al., 2018). This can involve comparing the rates of favorable or unfavorable decisions across different demographic groups, or using regression analysis to identify the factors that contribute most to the observed disparities.

Another important approach is to conduct algorithmic audits, which involve a systematic examination of the algorithm’s design, implementation, and use to identify potential sources of bias (Raji et al., 2020). This can include analyzing the training data for representational biases, testing the algorithm for differential performance across different subgroups, and reviewing the feature selection and weighting process for potential discriminatory effects. Algorithmic audits can be conducted internally by the organizations developing and deploying the systems, or externally by independent auditors or regulatory bodies.

However, detecting unintentional discrimination is only the first step; effectively regulating and mitigating it presents additional challenges. One key issue is the opacity of many algorithmic systems, which can make it difficult to understand how they arrive at their decisions and to identify the specific factors contributing to discriminatory outcomes (Burrell, 2016). This lack of transparency can hinder efforts to hold algorithms accountable and to develop targeted interventions to reduce bias.

Another challenge is the potential for algorithmic discrimination to be perpetuated or amplified through feedback loops, where the outputs of the algorithm are used to make decisions that then become inputs for future iterations, reinforcing existing biases (O’neil, 2017). For example, if an algorithm used to predict job performance consistently rates women lower than men, leading to fewer women being hired or promoted, this can create a self-fulfilling cycle that entrenches gender disparities over time.

To address these challenges, a range of regulatory approaches have been proposed and implemented in different contexts. One key strategy is to require algorithmic transparency and explainability, so that the basis for algorithmic decisions can be understood and scrutinized (Selbst and Powles, 2018). This can involve requiring companies to disclose the data and models used by their algorithms, as well as providing clear explanations of how individual decisions are made. The GDPR’s provisions on the right to explanation for automated decisions are an example of this approach.

Another important regulatory tool is impact assessments, which require organizations to proactively assess the potential discriminatory effects of their algorithmic systems and to take steps to mitigate any identified risks (Reisman et al., 2018). This can involve conducting algorithmic audits, as well as engaging with affected communities to understand their concerns and perspectives. The Canadian government’s Algorithmic Impact Assessment tool is an example of this approach, providing a standardized framework for assessing the risks and benefits of automated decision systems.

Other regulatory strategies include requiring regular monitoring and reporting on the outcomes of algorithmic systems, mandating the use of bias detection and mitigation techniques in the development process, and providing mechanisms for individuals to challenge or appeal algorithmic decisions that they believe are discriminatory (Kroll et al., 2017).

There have been some notable examples of effective management of unintentional algorithmic discrimination in recent years. For instance, in 2018, the U.S. Department of Housing and Urban Development (HUD) reached a settlement with Facebook over allegations that its targeted advertising system allowed housing providers to discriminate based on protected characteristics like race, gender, and disability (Housing, 2005). As part of the settlement, Facebook agreed to overhaul its ad targeting system to prevent such discrimination, including by no longer allowing housing, employment, or credit ads to be targeted based on protected categories, and by creating a separate portal for such ads with additional anti-discrimination safeguards.

Another example is the work of the Algorithmic Justice League, an organization that combines research, policy advocacy, and public engagement to raise awareness of algorithmic bias and to develop strategies for mitigating it (Buolamwini and Gebru, 2018). One of their key initiatives is the Safe Face Pledge, which calls on organizations to commit to not using facial analysis technology that has not been thoroughly tested for accuracy and bias across different demographic groups. By creating public pressure and accountability around this issue, the Algorithmic Justice League has helped to spur greater attention to the risks of unintentional discrimination in facial recognition systems.

However, despite these examples of progress, unintentional algorithmic discrimination remains a significant and ongoing challenge. In many domains, such as criminal justice risk assessment, hiring algorithms, and healthcare decision support systems, concerns about bias and disparate impact persist, and there is still much work to be done to develop and implement effective regulatory frameworks (Chouldechova and Roth, 2020).

One key area for future research and policy development is around the concept of algorithmic fairness, and how to define and operationalize it in different contexts (Corbett-Davies and Goel, 2018). While there are various statistical measures of fairness that can be used to assess algorithmic outcomes, such as demographic parity or equalized odds, there is no clear consensus on which measures are most appropriate or how to balance competing priorities like accuracy and fairness. Developing more nuanced and context-specific approaches to algorithmic fairness, and building them into the regulatory frameworks governing algorithmic systems, will be an important priority in the years ahead.

Another important challenge is how to address the potential for algorithmic discrimination to intersect with and exacerbate existing social inequities and power imbalances (Northpointe, 2015). For example, if an algorithm used to allocate public housing systematically disadvantages low-income communities of color, this can compound the effects of historical and ongoing discrimination in housing and urban development. Regulating algorithmic systems in isolation may not be sufficient to address these deeper structural issues, and a more holistic and intersectional approach may be needed.

## 7 Case studies

To illustrate the real-world manifestations and consequences of algorithmic discrimination, we present two case studies that highlight different types of bias and the legal and regulatory responses they have elicited.

### 7.1 COMPAS: racial bias in criminal risk assessment

One prominent case of algorithmic discrimination is the use of the COMPAS (Correctional Offender Management Profiling for Alternative Sanctions) system in the U.S. criminal justice system. COMPAS is a risk assessment tool developed by Northpointe (now Equivant) that predicts a defendant’s likelihood of recidivism based on a set of demographic, criminal history, and personal characteristics (Northpointe, 2015).

In 2016, a ProPublica investigation revealed that COMPAS exhibited significant racial bias in its predictions (Angwin et al., 2022). The analysis showed that the algorithm was more likely to falsely label Black defendants as high-risk for recidivism, while white defendants were more likely to be falsely labeled as low-risk. This disparate impact raised serious concerns about the fairness and validity of using algorithmic risk assessments in criminal sentencing and parole decisions.

The COMPAS case sparked a national debate on the role of algorithms in the criminal justice system and the need for greater transparency and accountability in their use. In State v. Loomis (2016), the Wisconsin Supreme Court ruled that the use of COMPAS in sentencing did not violate due process rights, as long as the algorithm’s limitations and potential biases were disclosed and it was not the sole determinant of the decision.

However, critics argued that the court’s decision did not go far enough in addressing the inherent biases and opacity of algorithmic risk assessments (Freeman, 2016). The case highlighted the challenges of relying on algorithms in high-stakes decision-making contexts and the need for more rigorous testing, auditing, and oversight of these systems.

### 7.2 Amazon’s hiring algorithm: gender bias in recruitment

Another notable case of algorithmic discrimination occurred in Amazon’s use of a machine learning system to screen job applicants. In 2018, it was revealed that Amazon had been developing an AI-powered recruiting tool to identify top candidates based on patterns in resumes submitted to the company over a 10-year period (Dastin, 2022).

However, the algorithm was found to exhibit significant gender bias, systematically penalizing resumes that included the word “women’s” or the names of women’s colleges. This bias stemmed from the historical underrepresentation of women in the tech industry and the fact that the training data predominantly consisted of resumes from male applicants. Amazon ultimately scrapped the project after attempts to mitigate the bias proved unsuccessful. The case underscored the risks of relying on historical data to train algorithmic systems, as it can perpetuate and amplify existing societal biases and inequalities.

The Amazon case also raised questions about the legal responsibilities of employers in preventing algorithmic discrimination in hiring. Under Title VII of the Civil Rights Act (Civil Rights Act of 1964, 1964), employers can be held liable for disparate impact discrimination, even if it results from seemingly neutral practices or algorithms. This highlights the need for proactive measures to audit and test algorithmic hiring systems for potential biases and to ensure compliance with anti-discrimination laws. These case studies demonstrate the pervasive and multifaceted nature of algorithmic discrimination and the challenges it poses for legal and regulatory frameworks. They underscore the importance of interdisciplinary research, public scrutiny, and policy interventions to promote greater fairness, transparency, and accountability in algorithmic decision-making systems.

### 7.3 Comparative analysis of algorithmic discrimination regulation

Algorithmic discrimination is a global issue that extends beyond the United States, and many countries and regions have developed their own legal and policy frameworks to address this challenge. In this section, we examine how algorithmic bias is being regulated in other jurisdictions and compare their approaches to those of the United States.

One notable example is the European Union’s General Data Protection Regulation (GDPR), which came into effect in 2018. The GDPR includes specific provisions related to automated decision-making and profiling, which are particularly relevant to algorithmic discrimination. Under Article 22 of the GDPR, individuals have the right to object to purely automated decisions that significantly affect them, and companies must provide meaningful information about the logic involved in such decisions (Goodman and Flaxman, 2017). This requirement for transparency and explicability goes beyond what is currently mandated in the United States, where there is no general right to an explanation for algorithmic decisions (Selbst and Powles, 2018).

In Canada, the proposed Algorithmic Accountability Act (Bill C-27) aims to regulate the use of automated decision-making systems by federal government departments and private sector organizations (Government of Canada, 2022). The bill requires companies to assess the risks of their automated systems, including potential biases and discriminatory outcomes, and take steps to mitigate those risks. This proactive approach to algorithmic accountability is similar to the preventive control measures discussed in the U.S. context but would be codified into law.

Australia has also taken steps to address algorithmic discrimination through its AI Ethics Framework, released in 2019 (Australian Government, 2019). The framework provides principles and guidance for the responsible development and use of AI systems, emphasizing fairness, non-discrimination, and accountability. While not legally binding, the framework encourages organizations to consider the potential for algorithmic bias and take steps to mitigate it throughout the AI lifecycle.

In Asia, countries like China, Japan, and South Korea have also recognized the importance of addressing algorithmic discrimination. For example, China’s New Generation Artificial Intelligence Development Plan, released in 2017, calls for the development of laws and regulations to ensure the safe and responsible use of AI, including measures to prevent discrimination (China State Council, 2017). Japan’s AI Utilization Guidelines, published in 2019, emphasize the need for fairness, accountability, and transparency in AI systems (Government of Japan, 2019).

These examples demonstrate that algorithmic discrimination is a concern shared by many countries and that there is a growing trend towards developing legal and ethical frameworks to address it. However, the specific approaches taken vary depending on the legal, cultural, and political contexts of each jurisdiction.

Compared to the United States, the European Union’s GDPR provides a more comprehensive and legally enforceable framework for algorithmic accountability, with explicit requirements for transparency and individual rights. Canada’s proposed Algorithmic Accountability Act also goes further than current U.S. regulations in mandating risk assessments and mitigation measures for automated decision-making systems.

However, the U.S. has a well-established legal framework for addressing discrimination through civil rights laws, which can be applied to algorithmic bias cases. The U.S. also has a strong tradition of judicial review and case law that can adapt to new technological challenges, as demonstrated by the cases discussed in Section 7.

Overall, this comparative analysis highlights the need for a multi-faceted approach to regulating algorithmic discrimination that combines legal requirements, ethical guidelines, technical standards, and ongoing monitoring and enforcement. While the specific mix of these elements may vary across jurisdictions, the goal of ensuring fairness, accountability, and transparency in algorithmic decision-making is a common thread.

As the use of AI and automated systems continues to grow globally, it will be increasingly important for countries to learn from each other’s experiences and best practices in addressing algorithmic discrimination. International collaboration and harmonization efforts, such as the OECD Principles on Artificial Intelligence (OECD, 2019a,b), can help to promote a more consistent and effective approach to this challenge across borders.

By incorporating this comparative analysis, our paper provides a more comprehensive and globally relevant examination of algorithmic discrimination and its regulation. It highlights the need for researchers, policymakers, and practitioners to consider the international dimensions of this issue and to work towards solutions that can be applied across different legal and cultural contexts.

## 8 Conclusion

This paper has provided a comprehensive overview of the types of algorithmic discrimination and the legal and regulatory approaches to addressing them, with a particular focus on the United States. By drawing on a systematic literature review, legal document analysis, and comparative case studies, we have developed a nuanced understanding of the challenges and opportunities for ensuring fairness and accountability in algorithmic decision-making.

Our analysis has shown that algorithmic discrimination can take many forms, from bias embedded in the data and features used by algorithms to more subtle and unintentional forms of disparate impact. We have identified five main types of algorithmic discrimination: bias by algorithmic agents, discrimination based on feature selection, proxy discrimination, disparate impact, and targeted advertising. Each of these types presents distinct challenges for detection, regulation, and remediation.

To address these challenges, we have examined the current legal and regulatory landscape in the United States, including principled and specific regulations, preventive controls, consequential liability, self-regulation, and heteronomy regulation. Our analysis suggests that while there are important legal and regulatory tools available for addressing algorithmic discrimination, there are also significant gaps and limitations in current approaches. In particular, we have highlighted the need for more proactive and preventive measures, such as algorithmic impact assessments and audits, as well as stronger transparency and accountability requirements.

Our comparative analysis of algorithmic discrimination regulation in other jurisdictions, including the European Union, Canada, and Australia, has revealed both common challenges and promising practices. While the specific legal and regulatory approaches vary across contexts, there is a growing consensus on the need for a multi-faceted and adaptive approach that combines legal requirements, technical standards, and ongoing monitoring and enforcement.

Based on our findings, we offer several recommendations for future research and policy development. First, there is a need for more interdisciplinary research that brings together computer science, law, social science, and ethics to develop a more holistic understanding of algorithmic discrimination and its social and legal implications. This research should focus on developing new methods for detecting and measuring bias, as well as evaluating the effectiveness of different regulatory and technical interventions.

Second, policymakers should prioritize the development of more comprehensive and proactive regulatory frameworks for algorithmic decision-making, drawing on best practices from around the world. This should include requirements for algorithmic transparency, impact assessments, and auditing, as well as stronger enforcement mechanisms and remedies for individuals and groups affected by discriminatory outcomes.

Third, there is a need for greater public awareness and engagement around the issues of algorithmic discrimination and fairness. This includes efforts to promote digital literacy and critical thinking skills, as well as opportunities for affected communities to participate in the design, development, and governance of algorithmic systems.

Finally, we emphasize the importance of ongoing monitoring and evaluation of algorithmic systems and their social and legal impacts. As the use of algorithms continues to expand across domains and jurisdictions, it will be essential to develop adaptive and responsive frameworks for ensuring their fairness, accountability, and transparency. This will require collaboration and coordination among researchers, policymakers, industry leaders, and civil society organizations.

In conclusion, algorithmic discrimination presents a complex and evolving challenge for society and the law. While there are no easy solutions, this paper has aimed to provide a comprehensive and nuanced analysis of the issues and to offer some potential pathways forward. By taking a proactive, interdisciplinary, and globally-engaged approach, we can work towards a future in which algorithmic systems promote rather than undermine social justice and equality.

## Data availability statement

The original contributions presented in the study are included in the article/supplementary material, further inquiries can be directed to the corresponding author.

## Author contributions

XW: Formal analysis, Investigation, Methodology, Writing – original draft. YW: Data curation, Investigation, Writing – original draft. XJ: Investigation, Methodology, Visualization, Writing – original draft. HF: Conceptualization, Project administration, Resources, Writing – review & editing.
